# Fish Granzyme A Shows a Greater Role Than Granzyme B in Fish Innate Cell-Mediated Cytotoxicity

**DOI:** 10.3389/fimmu.2019.02579

**Published:** 2019-10-31

**Authors:** Elena Chaves-Pozo, Yulema Valero, Maria Teresa Lozano, Pablo Rodríguez-Cerezo, Liang Miao, Vittorio Campo, Maria Angeles Esteban, Alberto Cuesta

**Affiliations:** ^1^Oceanographic Center of Murcia, Instituto Español de Oceanografía (IEO), Murcia, Spain; ^2^Fish Innate Immune System Group, Department of Cell Biology and Histology, Faculty of Biology, Campus Regional de Excelencia Internacional “Campus Mare Nostrum”, University of Murcia, Murcia, Spain; ^3^School of Marine Science, Ningbo University, Ningbo, China; ^4^Department of Chemical, Biological, Pharmaceutical and Environmental Sciences, University of Messina, Messina, Italy

**Keywords:** granzymes, cell-mediated cytotoxicity, granzyme A, granzyme B, nodavirus, fish

## Abstract

Granzymes (Gzm) are serine proteases, contained into the secretory granules of cytotoxic cells, responsible for the cell-mediated cytotoxicity (CMC) against tumor cells and intracellular pathogens such as virus and bacteria. In fish, they have received little attention to their existence, classification or functional characterization. Therefore, we aimed to identify and evaluate their functional and transcriptomic relevance in the innate CMC activity of two relevant teleost fish species, gilthead seabream and European sea bass. Afterwards, we wanted to focus on their regulation upon nodavirus (NNV) infection, a virus that causes great mortalities to sea bass specimens while seabream is resistant. In this study, we have identified genes encoding GzmA and GzmB in both seabream and sea bass, as well as GzmM in seabream, which showed good phylogenetic relation to their mammalian orthologs. In addition, we found enzymatic activity related to tryptase (GzmA and/or GzmK), aspartase (GzmB), metase (GzmM), or chymase (GzmH) in resting head-kidney leucocytes (HKLs), with the following order of activity: GzmA/K ~ GzmM >> GzmH >>> GzmB. In addition, during innate CMC assays consisting on HKLs exposed to either mock- or NNV-infected target cells, though all the granzyme transcripts were increased only the tryptase activity did. Thus, our data suggest a high functional activity of GzmA/K in the innate CMC and a marginal one for GzmB. Moreover, GzmB activity was detected into target cells during the CMC assays. However, the percentage of target cells with GzmB activity after the CMC assays was about 10-fold lower than the death target cells, demonstrating that GzmB is not the main inductor of cell death. Moreover, in *in vivo* infection with NNV, *gzm* transcription is differently regulated depending on the fish species, genes and tissues. However, the immunohistochemistry study revealed an increased number of GzmB stained cells and areas in the brain of seabream after NNV infection, which was mainly associated with the lesions detected. Further studies are needed to ascertain the molecular nature, biological function and implication of fish granzymes in the CMC activity, and in the antiviral defense in particular.

## Introduction

Granzymes (Gzm), formerly identified in the cytolytic granules of cytotoxic T lymphocytes (CTLs) and natural killer (NK) cells, belong to a family of serine proteases involved in the cell-mediated cytotoxicity (CMC or CMC activity), the major immune response against tumor cells and virus-infected cells (1–3). Later, it has been also demonstrated that they exert a role against bacterial infection (4, 5). Apart from CTLs and NK cells other cell types such as dendritic cells, B lymphocytes, mast cells or even non-immune cells are also able to produce Gzms. Upon CTL and NK cell activation, they release the content of the cytotoxic granules, perforin (PRF) and Gzms amongst others, and Gzms are able to enter and/or induce target cell death, mainly by apoptosis (6, 7). Granzymes are classified according to their functional enzymatic activity (related to humans): tryptase (GzmA and GzmK), aspartase (GzmB), metase (GzmM), or chymase (GzmH). GzmB and GzmA are the best characterized in human/mouse, being GzmB the most relevant for the CMC activity against tumor or virus-infected cells in humans (1, 2). Further characterization of the mammalian Gzm function and regulation during CMC is underway and still necessary.

Fish are the first group of vertebrates with a complete immune response possessing leucocytes involved in either innate or acquired CMC. Innate CMC is played by non-specific cytotoxic cells (NCCs), which express the NCC receptor protein-1 (NCCRP-1) marker and are morphologically variable, and/or NK-like cells, which do not express NCCRP-1 and are morphologically equivalent to the mammalian NK cells (8, 9). By contrast, fish CTLs express the T-cell receptor (TCR) and CD8 co-receptor, in a similar way than mammalian CTLs, and mediate the specific or acquired CMC (10, 11). Unfortunately, very little is known about the presence and role of the executor cytotoxic molecules, such as perforin or granzymes, in fish. First descriptions in fish demonstrated that, at least in part, the CMC activity is a granule-dependent mechanism, needs Ca^2+^ and a functional cytoskeleton, as in mammals, but very little has been described about perforin or granzymes at functional level (12, 13). The first molecular description of Gzms was performed in catfish (*Ictalurus punctatus*) immune tissues and NCCs by the identification of a Gzm-like mRNA sequence (named CFGR-1), which codes for a protein with 53 and 55% of similarity to human GzmA and GzmK, respectively (14). This finding was followed by genetic identification of several Gzms in different fish species (15–18). However, the low similarity of fish Gzms with their mammalian orthologs has led to ambiguous identification, classification and nomenclature. Due to this, some authors have proposed different nomenclature to fit them into a more general phylogenetic classification (15, 16, 18), though this has not been clearly adopted by the scientific community and adds even more controversy to the field.

Based on the scarce data about the proteolytic activity, differences have been observed in fish Gzms. The CMC activity of ginbuna crucian carp (*Carassius auratus langsdorfii*) leucocytes was almost completely abrogated by incubation with serine inhibitors, but not affected by cysteine inhibitors, demonstrating a major role of Gzms (19, 20). In addition, the use of GzmB specific inhibitors only reduced the CMC activity to 50%, suggesting the implication of several other granzymes (19). In catfish NCCs, the highest proteolytic activity during CMC assays was the chymase activity, followed by tryptase, and very low aspartase and metase activities (21). This suggests the highest role for GzmH in catfish, followed by GzmA/K, and lowest for GzmB and GzmM. However, they also showed that tryptase activity (GzmA/K) was the only one that greatly correlated to the innate cytotoxic activity (21). By contrast, allosensitized ginbuna crucian carp leucocytes only showed relevant tryptase activity during specific CMC activity (20). Gzm studies have also included the production of recombinant proteins from deduced mRNA sequences and the characterization of their proteolytic activity. Thus, recombinant catfish CFGR-1, catfish granzyme-like I and Nile tilapia (*Oreochromis niloticus*) TLGR-1 showed tryptase, metase, and chymase activity, respectively (21–23). However, when the mRNA sequence from ginbuna crucian carp, annotated as GzmB according to the sequence and phylogenetic analysis, was produced recombinantly it failed to show any conventional proteolytic activity against standard Gzm substrates (24). In addition, isolated ginbuna crucian carp GzmA has tryptase activity that is inhibited by serine inhibitors (20).

Granzymes also have an important role against viral infections since cytotoxic cells are responsible for the destruction of virus-infected cells. In this regard, viral infections trigger an increase on fish CMC activity and the expression of CMC-related genes (25). Nervous necrosis virus (NNV; *Nodaviridae* family, *Betanodavirus* genus) is the most serious pathogen for marine fish species, producing alterations in the central nervous system (brain and retina) with lethal consequences. NNV mainly affects to larvae and juvenile stages of fish. While European sea bass (*Dicentrarchus labrax*) is amongst the most susceptible fish species gilthead seabream (*Sparus aurata*) is refractory to almost all the NNV isolates, acting as a reservoir of the virus (26, 27). In addition, we have already demonstrated that both seabream and sea bass specimens infected with NNV showed increased NCC activity of head-kidney leucocytes (HKLs) against xenogeneic cells (28). Strikingly, isolated naïve sea bass HKLs are unable to kill fish target cells when they are infected with NNV (29). Therefore, we aimed with this study to detect and characterize the role of granzymes in gilthead seabream and European sea bass CMC response, with especial focus on GzmB, and how they are regulated by NNV. These data will throw some light on the evolution of granzymes, their role in the fish CMC response and how the NNV infection regulates it.

## Materials and Methods

### Animals

Adult individuals of the marine teleost gilthead seabream (*S. aurata*) and European sea bass (*D. labrax*) were bred at the Aquaculture facilities of *Mazarrón* of the *Instituto Español de Oceanograf*í*a* (IEO). Fish were transported to the University of Murcia and housed in 450–500 L running seawater (28‰ salinity) aquaria at 24 ± 2°C with a 12 h light:12 h dark photoperiod during 15 days prior to the experiments. Through all the time fish were fed daily with 2 g per fish using a commercial pellet diet (Skretting). Animal handling and sampling was approved by the Bioethical Committees of the IEO and the University of Murcia (Permit Number: A13150104). All the *in vitro* assays followed the general guidelines for Good Practice Laboratory principles.

### Fish Cell Lines, Leucocytes, and Virus

The established fish cell line E-11, derived from the SSN-1 cell line from striped snakehead (*Channa striatus*), was used (30). Cells were incubated at 25°C in an atmosphere with 85% relative humidity using L-15 Leibowitz medium (Life Technologies) supplemented with 10% fetal bovine serum (FBS, Life Technologies), 2 mM L-glutamine (Life Technologies), streptomycin 100 μg/mL (Life Technologies) and penicillin (100 U/mL, Life Technologies). Cells were subcultured by routine trypsinization and centrifuged at 400 g for 10 min. Cells were counted and viability higher than 95% as determined by the trypan blue staining.

For isolation of resting head-kidney leucocytes (HKLs), gilthead seabream and European sea bass specimens were bled and after dissection the head-kidney was cut into small fragments and transferred to L-15 culture medium containing 5% FBS, L-glutamine, penicillin and streptomycin. Tissue fragments were pressed to be disorganized and passed by 100 μm nylon mesh to obtain cellular suspensions. Cells were washed twice, counted and adjusted to 10^7^ cells/mL. Viability resulted higher than 95% by the trypan blue staining.

Nodavirus (strain It/411/96, genotype RGNNV) were produced using E-11 as host cells at 25°C until the cytopathic effect was extensive. Supernatant was obtained after centrifugation and viral titer determined in 96-well plates before used in the experiments (31).

### Gene Sequence Analysis

Sea bass genome (http://seabass.mpipz.mpg.de/), an internal seabream RNA-seq and GenBank EST databases were searched for the identification of granzyme genes. This resulted in the annotation of seabream *gzma, gzmb*, and *gzmm*, as well as sea bass *gzma* and *gzmb*. All the sequences were analyzed for similarity with other known sequences using the BLAST program (32) within the ExPASy Molecular Biology server (http://us.expasy.org), which was also used to predict the protein structure. Multiple sequence alignments were carried out using the CLUSTALW program, within the European Bioinformatics Institute. Phylogenetic and molecular evolutionary analyses were conducted using MEGA version 6 (33). A phylogenetic tree was constructed using the identified putative protein sequences with other fish and human/mouse granzymes.

### Granzyme Characterization in *in vitro* CMC Assays

CMC assays were performed to determine either innate CMC activity, Gzm activity, or *gzm* gene expression studies, as well as to evaluate the role of GzmB.

#### CMC Assays

E-11 cells were seed in 96-well bottomed-flat plates (Nunc) at a density of 15,000 cells/well without (mock) or with 10^6^ TCID_50_ NNV/mL (NNV-infected) and used as targets. After 24 h of incubation at 25°C, wells were washed with culture medium and 100 μL of isolated HKLs (effectors) were added at an approximate ratio of 50 HKLs per target cell. Samples were then centrifuged at 400 g for 1 min to favor cellular contact and incubated for 4 h at 25°C. The experiment was conducted using HKLs from five individual fish in separate and each sample was done in triplicate. E-11 cell infection with NNV was confirmed by real-time PCR (qPCR) (29).

#### Innate CMC Activity by Flow Cytometry

Mock- or NNV-infected target cells were labeled with 5 μM CFSE (carboxyfluorescein succinimidyl ester; Sigma-Aldrich) for 15 min (34), to be distinguished to the leucocytes (CFSE^−^) during flow cytometry analysis, washed, counted, and used in the CMC assays as above. After 4 h of incubation with HKLs at 25°C, cells were detached, propidium iodide (PI; Sigma-Aldrich) added at 40 μg/mL and samples acquired and analyzed in a flow cytometer (FACSCalibur, Becton Dickinson) set to analyze the target cells (CFSE^+^PI^−^; alive; CFSE^+^PI^+^, death cells). CMC samples incubated for 0 min served as controls or blanks. Activity was calculated by the following formula:

$$\begin{array}{l} \text{CMC activity }\left(\text{\%}\right)=100\times \\ \left[\left(\text{CMC sample }-\text{ CMC blank}\right)/\left(100-\text{CMC blank}\right)\right] \end{array}$$

#### Gene Expression

CMC samples were centrifuged, TRIzol Reagent (Life Technologies) added and immediately stored at −80°C for latter RNA isolation. Gene expression was evaluated by qPCR as described below.

#### Granzyme Activity

Granzyme activities were determined using colorimetric methods. Reaction buffer [50 mM Tris–HCl, 0.15 M NaCl, 0.01% Triton X-100 (pH 7.6) containing 0.2 mM Ellman's reagent (Sigma-Aldrich)] was added to CMC samples and cells lysed with extensive vortexing. Samples consisting on target cells or HKLs alone were used as controls. For this, 10 μL of samples were dispensed in 96-well plates and mixed with 90 μL of reaction buffer containing 0.2 mM of the corresponding specific substrates: Z-Lys-SBzl (Sigma-Aldrich) for tryptase/GzmA/K, N-Acetyl-Ile-Glu-Pro-Asp-p-nitroanilide (Ac-IEPD-pNA; Sigma) for aspartase/GzmB, Boc-Ala-Ala-Met-SBzl (MP Biochemicals) for metase/GzmM or N-Succinyl-Ala-Ala-Pro-Phe p-nitroanilide (Suc-AAPF-pNA; Sigma) for chymase/GzmH activities. Substrates were dissolved in dimethyl sulfoxide (DMSO) and stored at −20°C. Optical density (OD) was determined at 405 nm at 0 and 90 min of incubation being the OD change determined. Reaction buffer replaced the sample in blanks.

#### Role of GzmB in CMC Assays

Mock- or NNV-infected target cells were labeled with the TLF4 reagent from the GranToxiLux® PLUS kit (OncoImmunin). After exhaustive washing, seabream or sea bass HKLs were added, centrifuged at 400 g for 1 min and the GS reagent (Granzyme B substrate; excitation 485 nm, emission 520 nm) added. After 90 min of incubation samples were analyzed in a flow cytometer set to distinguish effector (non-fluorescent) and the target (TLF4^+^) cells. Target cells were then selected and dot-plots against the granzyme B activity (GS^+^, FL1) were studied to determine the percentage of target cells with granzyme B activity (TLF4^+^FL1^+^) in the CMC assays.

### Fish Infection With Nodavirus

Gilthead seabream (125 ± 25 g) or European sea bass (305 ± 77 g) specimens were allocated in four aquaria of 450–500 L with a closed-recirculation seawater system at 24 ± 2°C, 12 h light:12 h dark photoperiod and fed with a commercial diet. After 2 weeks of acclimation, fish from each species received an intramuscular injection with culture medium alone (mock- or controls) or NNV (10^6^ NNV TCID_50_/fish) (28). Five fish were sampled at 1, 7, or 15 days after the viral injection. HK and brain tissues were sampled in TRIzol Reagent for latter RNA isolation and gene expression by qPCR while seabream brain tissue was processed for immunohistochemistry (IHC).

### Real-Time PCR (qPCR) for Gene Expression

Total RNA from CMC assays, or HK and brain from *in vivo* infections, was isolated following manufacturer instructions. Genomic DNA was removed by DNase I (Promega) digestion and cDNA synthetized by SuperScript III reverse transcriptase (Thermo Scientific). qPCR was performed with an ABI PRISM 7500 instrument (Applied Biosystems) using SYBR Green PCR Core Reagents (Applied Biosystems) as elsewhere (28). mRNA transcription was corrected by the elongation factor 1-alpha (*ef1a*) content in each sample and expressed as 2^−Δ*Ct*^ (35). The primers used are shown in Table 1.

**Table 1:** Primer sequences used for real-time PCR (qPCR).

**Fish species**	**Gene name**	**Acc. number**	**Primer sequences**
European sea bass	Granzyme A	KJ818347	TCCCTGCTATGATGCAACTG ATTTCACCGTCTTGGTTTGC
	Granzyme B	DLAgn_00151210	AAGTTGAGCTCCAAGGCAAA TCCCCAGCCAGAGATGATAC
	Elongation factor 1a	AJ866727	CGTTGGCTTCAACATCAAGA GAAGTTGTCTGCTCCCTTGG
Gilthead seabream	Granzyme A	MK568066	GCTGCTCGGAGTCACTTCTT GGATCCAGGTGAGCTGCTTT
	Granzyme B	AM957224	GAAACAAAGGAACGGGTCAA GAGCTGTCCATCTTTTGCTTG
	Granzyme M	MK568067	TCGGACGAAATGCACTTGGA ACATGTGTCCGTCTGTGGTC
	Elongation factor 1a	AF184170	CTTCAACGCTCAGGTCATCAT GCACAGCGAAACGACCAAGGGGA

### Immunohistochemistry (IHC)

The sequence of seabream GzmB protein was predicted and used for antigenic peptide evaluation, peptide synthesis (KINDAQRVSVEQAFC) and polyclonal antibody production. A rabbit polyclonal anti-seabream GzmB was produced (GenScript), which only showed reactivity for seabream with IHC technique.

In mock- or NNV-infected fish, the brain tissues were sampled and fixed in Bouin's solution for 16 h at 4°C, embedded in paraffin (Paraplast Plus; Sherwood Medical) and sectioned at 5 μm. After dewaxing and rehydration, sections were incubated with anti-GzmB antiserum at 1:100 for 1 h and processed as elsewhere. Negative controls consisted on pre-immune serum, the lack of antiserum or in the pre-incubation of the antiserum with the synthetic peptide. Sections were also stained with hematoxylin and eosin (HE). Slides were examined in an Axiolab (Zeiss) light microscope and photographed.

### Statistical Analysis

Figures are presented as mean ± SEM (*n* = 5) of the data. Statistical differences between groups were analyzed by either *t*-Student or one-way analysis of variance (ANOVA; *P* ≤ 0.05) followed by the comparison of mean test of Tukey.

## Results

### Identification of Granzyme Transcripts

**Figure 1:**
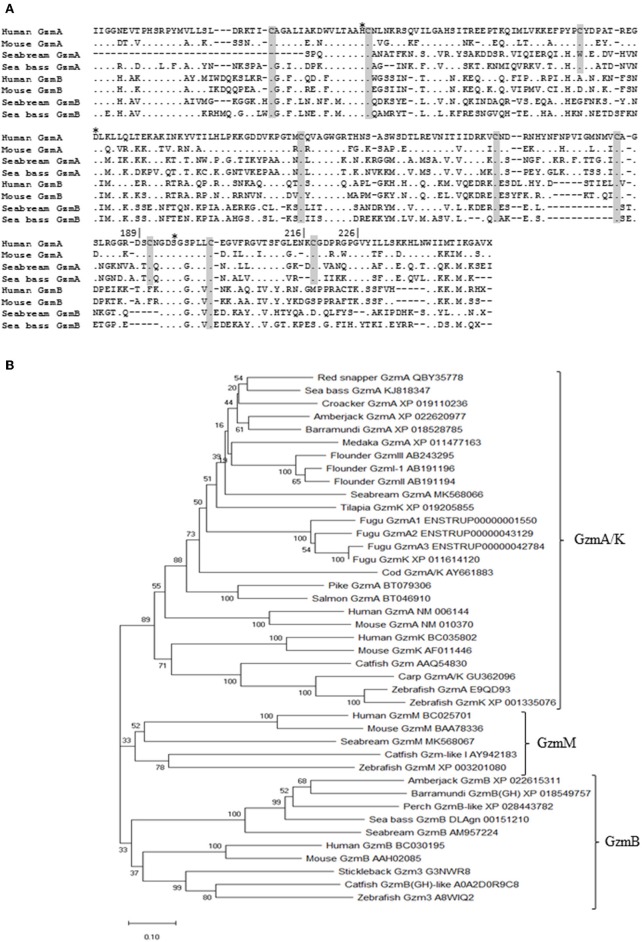
Identified seabream and sea bass granzymes are evolutionary conserved. **(A)** Mature peptide protein sequences of gilthead seabream, European sea bass, human and mouse GzmA, and GzmB were annealed. Dots represent conservation to the human GzmA. Gray boxes denote the conserved Cys and asterisks the catalytic triad residues. Numbers on top the sequence is based on the human chymotrypsin sequence. **(B)** Phylogenetic tree was constructed with protein sequences of the mature granzymes by the Neighbor-Joining method. Genetic distances were calculated based on protein differences (p-distance) with partial deletion option. The number at each node indicates the percentage of bootstrapping after 1,000 replications. Accession numbers for each sequence are indicated.

We have identified in the databases the mRNA sequences of *gzma* and *gzmb*, in both fish species, and *gzmm* only in seabream. The European sea bass *gzma* and *gzmb*, and the gilthead seabream *gzmb*, gene sequences were complete, while the seabream *gzma* and *gzmm* genes were partial and lacked the 5′- and 3′-end, respectively. Granzymes are produced as pre-pro-peptides with a leader peptide, being the pro-peptide excised after the first two amino acids releasing the very well conserved IIGG sequence in the N-terminus of the active granzymes. Protein sequences of seabream and sea bass GzmA and GzmB were annealed with their human and mouse orthologs showing amino acid identity of 40–50%, though key residues, such as those Cys involved in disulphide bonds and the catalytic triad, are very well conserved (Figure 1A). In addition, a phylogenetic tree was constructed including active Gzm sequences from other fish species and human and mouse (Figure 1B). The three identified Gzms appeared in different clades together to fish and mammalian orthologs.

### Granzyme Transcription and Innate CMC Activity Are Related

**Figure 2:**
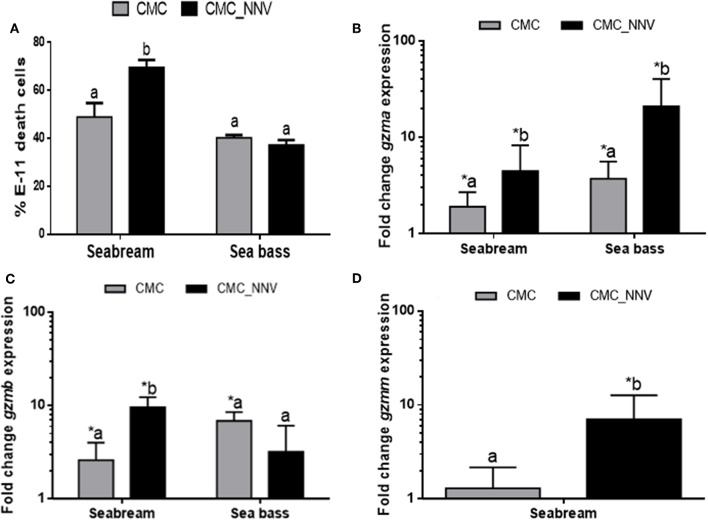
Cell-mediated cytotoxicity (CMC) and granzyme transcription are related**. (A)** The percentage of death E-11 target cells, either mock- (CMC) or NNV-infected (CMC_NNV), upon innate CMC activity of gilthead seabream and European sea bass leucocytes was determined by flow cytometry. Data represent the mean ± SEM (*n* = 5). Different letters denote differences between the CMC activity against mock- and NNV-infected targets (*t*-Student, *P* ≤ 0.05). **(B–D)** Transcription of the *gzma* and *gzmb* in both fish species, and *gzmm* in seabream was evaluated in CMC assays using either seabream or sea bass leucocytes against mock- (CMC) or NNV-infected (CMC_NNV) target cells. Data represent the mean ± SEM (*n* = 5) of fold change respect to leucocytes alone. Asterisks denote significant differences between the transcription respect to the control (leucocytes alone) while different letters denote differences between mock- and NNV-infected targets (*t*-Student, *P* ≤ 0.05).

After granzyme identification, their transcription was evaluated in HKLs during CMC assays against xenogeneic mock- or NNV-infected cells. As previously demonstrated (29), the innate CMC activity is significantly increased against NNV-infected target cells compared to mock- or non-infected cells in the case of seabream HKLs, but not for sea bass leucocytes (Figure 2A). Transcription of *gzma* and *gzmb*, but not *gzmm*, was significantly up-regulated in seabream HKLs against mock-infected target cells compared to naïve HKLs alone, and this up-regulation was even higher when the targets were infected with NNV (Figures 2B–D), similarly to the CMC activity (Figure 2A). In the case of sea bass HKLs, the expression of both *gzma* and *gzmb* genes was significantly up-regulated against E-11 target cells compared to naïve HKLs, however, only the mRNA levels of *gzma* were further increased during CMC assays against NNV-target cells (Figures 2B,C).

### Granzyme Activities Point to a Major Role of GzmA/K in the Innate CMC Activity

**Figure 3:**
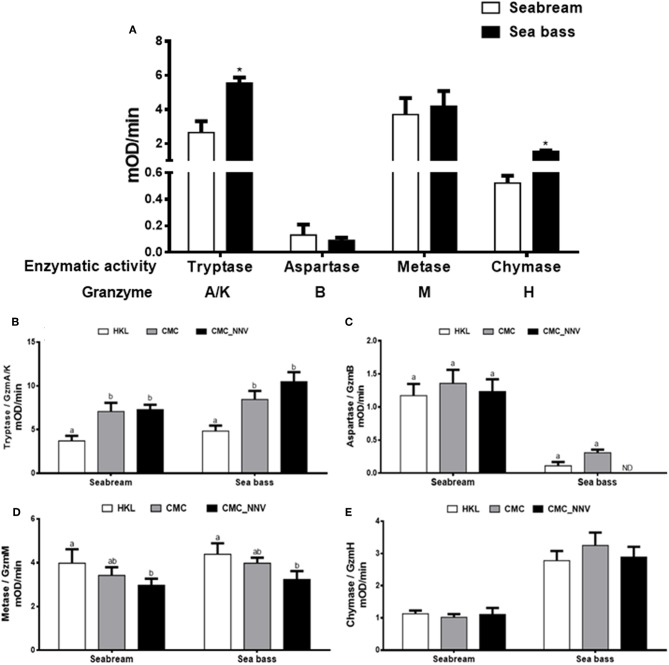
Fish leucocytes show granzyme activities being tryptase activity the highest during CMC reaction. **(A)** Gilthead seabream and European sea bass isolated head-kidney leucocytes (HKLs) were lysed and incubated with specific substrates to evaluate the tryptase, aspartase, metase, and chymase activities by colorimetry. Bars represent the mean ± SEM (*n* = 5). *t*-Student statistical differences between fish species are denoted at *P* ≤ 0.05 with asterisks. **(B–E)** Seabream and sea bass HKLs alone or in CMC assays against mock- (CMC) or NNV-infected (CMC_NNV) E-11 target cells were assayed for tryptase **(B)**, aspartase **(C)**, metase **(D)**, or chymase **(E)** activities. Bars represent the mean ± SEM (*n* = 5). Different letters denote significant differences based on the ANOVA and Tukey's comparison on means test at *P* ≤ 0.05.

Once the granzyme genes were identified we determined whether seabream or sea bass leucocyte lysates have tryptase, aspartase, metase, or chymase enzymatic activities, which are related to the human A/K, B, M, or H granzyme activities, respectively. Tryptase, metase, and chymase activities were clearly detected in both seabream and sea bass naïve HKL lysates, whilst the aspartase activity ranged from no detection to very low levels (Figure 3A). In addition, tryptase and chymase activities were significantly higher in sea bass HKLs than in seabream leucocytes, while the others showed very similar levels. During innate CMC assays with HKLs, our results show that the different granzyme activities are differently regulated (Figures 3B–E). First, tryptase activity (Figure 3B) was the unique granzyme activity significantly increased in HKLs incubated with E-11 target cells in both fish species, though this was not significantly different when the target cells were mock- or NNV-infected. Strikingly, aspartase activity was unaltered in seabream HKLs during CMC assays against target cells alone, but it was blocked in sea bass HKLs incubated with NNV-infected cells (Figure 3C). Metase activity was significantly decreased in the HKLs of both seabream and sea bass CMC assays against NNV-infected cells (Figure 3D). Finally, chymase activity was unaltered in HKLs during the CMC activity (Figure 3E).

### Fish CMC Activity *in vitro* Is Slightly Dependant on GzmB

**Figure 4:**
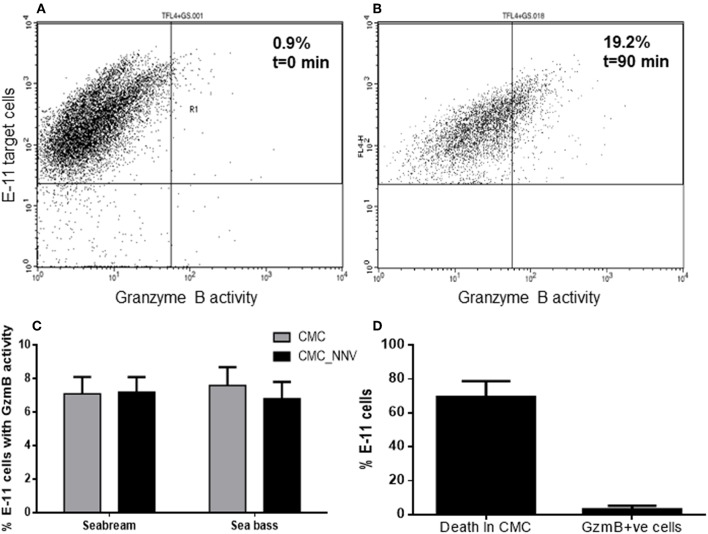
Granzyme B is incorporated into target cells but show scarce killing capacity. **(A,B)** Representative dot-plots showing the granzyme B activity (FL1^+^) into the E-11 target cells before **(A)** and upon incubation **(B)** with gilthead seabream head-kidney leucocytes. **(C)** Percentage of target cells, either mock- or NNV-infected, showing granzyme B activity after being incubated for 90 min with seabream or sea bass leucocytes. Bars represent the mean ± SEM (*n* = 5). **(D)** Representation of the percentage of death target cells and GzmB+ve target cells after the innate CMC activity played by seabream leucocytes. Bars represent the mean ± SEM (*n* = 5). CMC, cell-mediated cytotoxicity assays against mock-infected target cells; CMC_NNV, cell-mediated cytotoxicity assays against nodavirus-infected target cells.

As aspartase activity was hardly detected (Figure 3C) with the previous methodology, and thanks to the availability of a commercial kit, we re-evaluated the aspartase or GzmB activity released from fish HKLs and incorporated into the target cell population using flow cytometry and a more sensitive technique. We successfully detected GzmB activity into the E-11 target cells (TLF4^+^GS^+^) (Figures 4A,B) and quantified how the GzmB activity into the target cells was increased during CMC assays. The analysis of the percentage of E-11 cells with GzmB activity upon incubation with fish HKLs was very low and independent on the source of HKLs and on the presence of NNV infection (Figure 4C). The percentage of GzmB^+^ target cells is 10-fold lower than the percentage of death target cells (Figure 4D; see also Figures 4C, 2A), what suggests a marginal role of GzmB in the target cell killing by fish HKLs.

### Granzymes Are Differently Transcribed Upon NNV Infection *in vivo*

**Figure 5:**
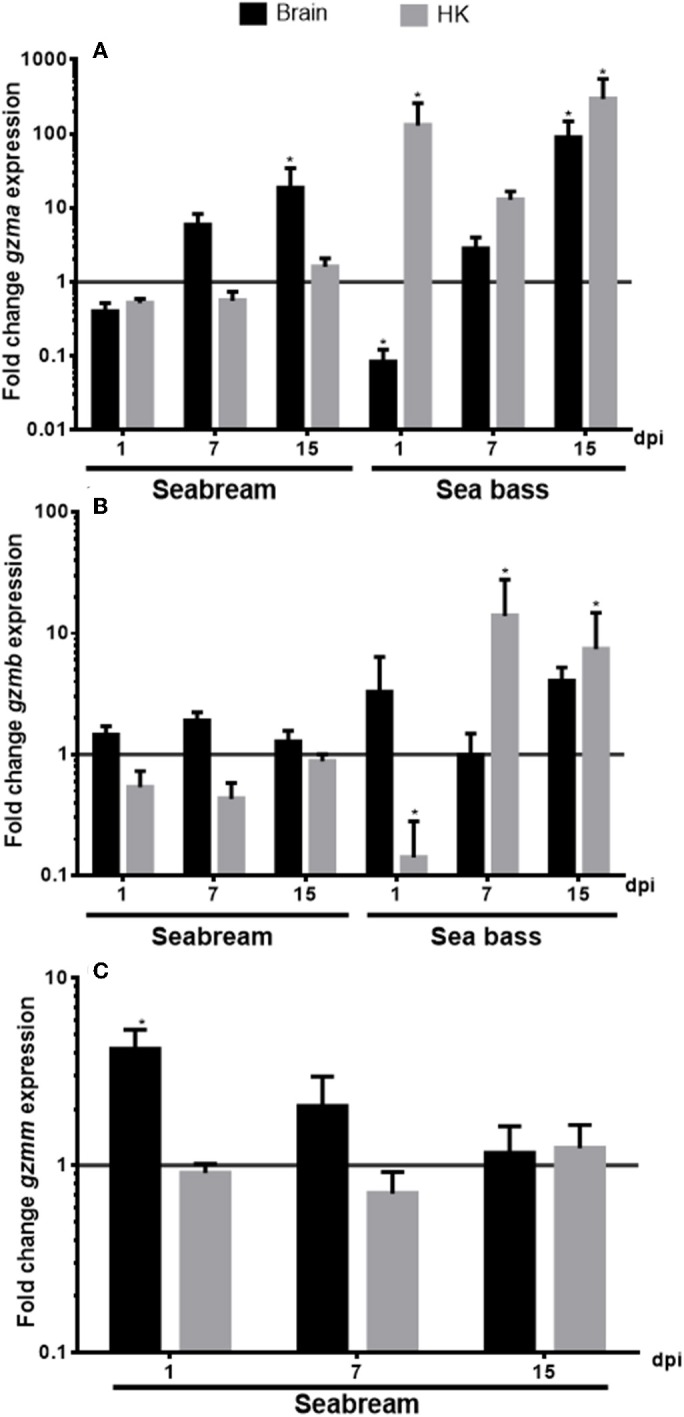
Nodavirus (NNV) infection up-regulated the transcription of granzymes. The gene expression of *gzma* **(A)**, *gzmb* **(B)**, and *gzmm* **(C)** was determined by qPCR in the brain or head-kidney (HK) from gilthead seabream or European sea bass specimens at 1, 7, or 15 days-post-infection (dpi) with nodavirus (NNV). Data represent the mean ± SEM (*n* = 5) of the fold change respect to the values found in controls. Asterisks denote significant differences between NNV-infected and controls (*t*-Student, *P* ≤ 0.05).

We evaluated the transcription of the identified granzyme genes in the brain and HK tissues from seabream and sea bass specimens during a NNV infection (Figure 5). In the case of the seabream HK, the transcription of any of the granzymes was significantly altered upon NNV infection. By contrast, in the seabream brain, the transcription of *gzma* (Figure 5A) was increasing with the infection time, reaching significance after 15 days of infection, whilst *gzmm* gene expression (Figure 5C) did the inverse, being significantly up-regulated after 1 day and decreasing thereafter. In the case of European sea bass, the NNV infection decreased the *gzma* gene expression after 1 day of infection in the brain, and continuously increased thereafter till 15 days, similar to the seabream *gzma* gene expression (Figure 5A). However, *gzma* transcription was always highly up-regulated in the HK of NNV-infected sea bass specimens. By contrast, the *gzmb* gene expression was decreased after 1 day of infection in the sea bass HK, and increased thereafter, while it was not altered in the brain (Figure 5B).

### GzmB Immunostained Cells and Areas Are Increased in Seabream Brain Upon NNV Infection

**Figure 6:**
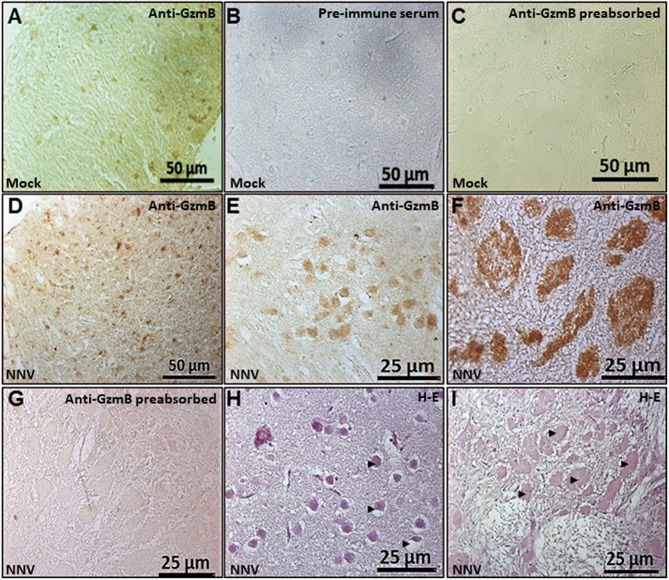
Number of GzmB positive cells and area increased in the seabream brain upon NNV infection. Representative sections of brain from gilthead seabream specimens mock- **(A–C)** or NNV-infected during 7 days **(D,E,H)** or 15 days **(F,G,I)** subjected to immunohistochemistry (IHC) using an anti-serum against seabream GzmB **(A**-**G)** or stained with hematoxylin-eosin **(H,I)**. Sections immunostained with anti-Gzm B serum **(A,D–F)**, with the pre-immune serum **(B)** or with pre-absorbed immune serum **(C,G)** are shown. Representative images of the histopathological features observed in the brain upon 7 **(H)** and 15 **(I)** days of NNV infection. Black arrow heads point to the necrotic areas of the brain. Mock, control; NNV, infected with nodavirus.

An in-house produced anti-seabream GzmB antiserum was used in the brain of seabream and sea bass by IHC but this was successful only for seabream. Based on the specific reactivity of the anti-seabream GzmB we were able to evaluate its presence in the seabream brain upon NNV infection (Figure 6). Results indicated the presence of this GzmB in some brain cells of control or non-infected seabream specimens (Figure 6A). The specificity of the antibody was determined due to a complete lack of reactivity when pre-immune serum was used (Figure 6B) and when the anti-serum was pre-incubated with the synthetic peptide (Figures 6C,G). The number of GzmB+ve cells and the staining areas were increased in seabream brain upon NNV infection (Figures 6D–F) compared to control fish (Figure 6A). The largest areas with specific reactivity corresponded to necrotic or degenerated areas as demonstrated by the routine histological observations (Figures 6H,I).

## Discussion

In humans, there are five granzymes with known activities: GzmA and GzmK have tryptase, GzmB has aspartase, GzmM has metase, and GzmH has chymase activity (1, 2). In fish, however, several granzymes and granzyme-like sequences have been identified after genomic or transcriptomic analysis (15, 16, 18) or enzymatic activity studies (14–16, 18–24). Although fish orthologs to mammalian Gzms have been clearly identified some studies have also dealt with important differences at genetic level. In fact, the low identity between fish and mammalian Gzms has generated great variability in terms of nomenclature that is hampering their characterization. Based on the mRNA sequences, gilthead seabream and European sea bass putative GzmA and GzmB have very well-conserved most of the characteristics of their mammalian orthologs. Thus, GzmA shows good conservation of key residues in both fish species, including the canonical catalytic triad for serine proteases (His_57_, Asp_102_, and Ser_195_), 8 Cys to form disulphide bonds as well as the Asp_189_, Gly_216_, and Gly_226_ involved in the substrate specificity pocket (18). The amino acids involved in this substrate specificity pocket, which are conserved with mammalian orthologs, have been found in several fish species. This issue has led to suggest that GzmA/K appeared back to 420 million years before the tetrapod divergence, and represent the first group of serine proteases involved in immunity (18). However, this substrate pocket has not been clearly identified in other granzymes. In addition, different criteria in the classification and nomenclature of fish serine proteases in general, and granzymes in particular, are leading to some obscurity at this regard. For example, the names of Gzm-like I and II in cod (*Gadus morhua*), or II in catfish (18, 23). Therefore, further studies are needed to unequivocally identify fish granzymes at genetic and structural levels.

Apart from the limited knowledge in the fish Gzms repertoire, their role and implication in the immune response, and in the CMC activity in particular, as well as their regulation, deserve deeper characterization. Thus, our data show that the tryptase and metase activities are the highest in naïve seabream and sea bass leucocytes, and aspartase the lowest. There is only one study at this respect in fish demonstrating that resting kidney leucocytes of ginbuna crucian carp show very low levels of aspartase, metase, or chymase activities, being tryptase activity the highest (20). Strikingly, some studies carried out in humans have demonstrated different levels of Gzms depending on the of presence of NK cell and CTL subpopulations. Similar to our data, human GzmA (tryptase activity) is the most expressed in resting peripheral blood (PBL) NK cells and CD8^+^ CTLs, with roughly similar levels of GzmB (aspartase activity), and much lower levels of GzmK (tryptase activity) (36). Another study also showed that the GzmM (metase activity) levels were similar to GzmA (37). Strikingly, GzmH (chymase activity) was shown to be similarly expressed than GzmB in whole human's PBLs, though GzmH was predominantly expressed in NK cells while GzmB did in NKT and CD8^+^ cell subpopulations (38). Thus, it is clear that apart from the leucocyte activation state the representation and maturity of the different cell populations are important factors when determining the total Gzm presence or activity. Unfortunately, this leucocyte-type classification is by far not possible for fish due to the lack of proper tools for leucocyte markers.

When leucocytes encounter an exogenous cell, xenogeneic or allogeneic, virus-infected or not, the CMC response is triggered and leucocytes kill the target cells by both apoptosis and necrosis, which is mediated by both granule-dependent and -independent mechanisms. Among the granule-dependent mechanisms, the involvement of perforin and granzymes are the most important and well-known. As in mammals, CMC activity mediated by fish NCC or NK-like is increased upon target cell contact, which is also related to the Gzm activity. Thus, in ginbuna crucian carp, allostimulation of kidney leucocytes resulted in increased tryptase, and reduced metase, activities, while isolated CD8^+^T PBLs showed increased aspartase activity (19, 20). In addition, the mRNA expression of the named *gcgzm* (most probably GzmB) gene was significantly up-regulated in CD8^+^ cells, but not in CD4^+^ or IgM^+^ cell populations (24). In catfish, the activity of chymase was the highest during the CMC response, followed by tryptase, aspartase, and metase (21). Our data also show that during CMC assays the *gzma* and *gzmb* transcription is up-regulated, though at functional level only the tryptase activity does, which is partly supporting the data found in crucian carp, and points to a major role of GzmA in fish innate CMC activity, with probably marginal roles of GzmB, GzmM, and GzmH. By contrast, human or mouse CMC activity is mainly mediated by GzmB, though GzmA has also a great contribution (1, 4, 39). To go deeper we also evaluated the potential relevance of fish GzmB on target cell killing. Thus, be means of a commercial kit we were able to characterize the presence of GzmB activity into target cells, which is known to be released from mammalian leucocytes, enter into the target cells and induce apoptosis cell death. Our data show that only few of the target cells are GzmB+ve, which represent around 10% of the total death target cells. This issue also supports the very low aspartase activity observed in our CMC assays. Thus, these findings imply that other granzymes might be the main mediators of this innate CMC activity, and this could be the GzmA, as our data of mRNA levels and activity suggest. A previous study evaluating the ginbuna crucian carp GzmB activity showed that aspartase activity is increased in CD8^+^ T cells, parallel to the specific CMC activity, but the use of specific GzmB inhibitors only reduced the CMC activity around 50%, supporting our observation that GzmB is not the main mediator of the fish CMC activity (19). Comparatively, in humans, GzmB, followed by GzmA, are the most induced granzymes in NK cells and/or CTLs upon lymphocyte activation though variable levels and different regulation have been observed at cell-to-cell levels, a fact the complicates even more the knowledge in the biology of granzymes (36–38). In addition, Gzms are known to induce apoptosis-like cell death by inducing several mechanisms. In fish, including seabream and sea bass, it is known that CMC activity led to target cell death by both necrotic and apoptotic pathways (40–42) though the precise molecular mechanisms are unrevealed. Unfortunately, nothing is known about how fish granzymes are released, enter the target cell, their molecular targets and the killing mechanisms they induce, what merits further characterization.

The immune response against virus is mainly mediated by the interferon (IFN) pathway and the CMC response, the latter being slightly evaluated in fish. When target cells are infected with virus the mammalian CMC activity is higher, when compared to the same target cell without virus, as also happens with the fish CMC activity against virus-infected cells (25). So, granzymes do not have antiviral activity *per se* but are responsible for the leucocyte killing of virus-infected cells. Regarding NNV, we have already demonstrated that the CMC activity of the resistant seabream HKLs against NNV-infected cell lines is significantly higher than against mock-infected cells (29), as also evidenced in this study. By contrast, HKLs isolated from the very susceptible European sea bass were not able to do so and this could be considered a fact that could partly explain this fish species susceptibility. Our present results about granzymes are not definitive at this respect. Both *gzma* transcription and/or tryptase/GzmA/K activity are increased in HKLs during CMC assays against NNV-infected target cells and up-regulated upon *in vivo* NNV infection in both fish species. Similarly, mRNA levels of *gzma* are also up-regulated in fish by viral infections: in common carp (*Cyprinus carpio*) by spring viremia of carp virus (SVCV) (17), in rainbow trout (*Oncorhynchus mykiss*) RTS-11 cell line and leucocytes by viral haemorrhagic septicaemia virus (VHSV) (43), in Atlantic salmon (*Salmo salar*) by infectious pancreatic necrotic virus (IPNV) (44, 45) or in sea bass retina by NNV (46). In addition, mRNA levels of Atlantic salmon and ginbuna crucian carp *gzma* were also up-regulated by bacterial infections (24, 45). Interestingly, the expression of *gzma* gene in Atlantic salmon was correlated with those of *cd8* gene and considered a good marker for acute IPNV infection (45). All these data point to an important role of GzmA during the CMC response against viral infections. The potential role of other granzymes in antiviral immunity is almost ignored in the literature.

Our data regarding GzmB are controversial since data about transcription profile, innate CMC, aspartase activity and GzmB localization and distribution during NNV infections are not well-correlated. In the CMC assays against NNV-infected cells, the increased *gzmb* transcription in seabream HKLs is parallel to the increased CMC activity, but not to the aspartase activity or the percentage of GzmB+ve target cells when compared to the mock-infected target cells. By contrast, in sea bass HKLs neither the mRNA or activity were increased. However, data from *in vivo* infection with NNV indicate that the *gzmb* transcription is up-regulated by NNV infection only in the HK of sea bass. This issue is also supported by a previous study in which the up-regulation of the *gzmb* mRNA levels in the retina of sea bass specimens upon NNV infection was reported (46). However, this is the first study evaluating the detection of fish granzymes at protein level. To do this, anti-seabream GzmB antiserum was obtained and satisfactorily applied in the IHC technique for seabream. Unfortunately, this antiserum failed to detect GzmB in the sea bass samples, probably because only 5 out of the 15 amino acids were conserved. This conservation was even lower for seabream GzmA or GzmM, and no cross-reaction is expected. Since transcription of *gzmb* was not increased in the brain of seabream upon *in vivo* infection but the IHC study reveals great increases in the number of GzmB+ve cells and in the size of the staining areas it is tentative to speculate that: (i) a post-transcriptional regulation occurs in the brain, (ii) the regulation of the CMC activity and its mediators is different *in vitro* and *in vivo*, (iii) there is a local and specific regulation in the brain since it is known that immune-privileged tissues have a different pattern of regulation than proper immune tissues (47), or (iv) seabream lymphocytes migrate from the thymus or HK and infiltrate into the brain. A previous work also described the infiltration of IgM^+^ cells in the brain of seabream upon NNV infection, but not of macrophages or acidophilic granulocytes (48). These two observations, together with the increase of the transcription level of lymphocyte marker genes in the brain after NNV infection (48–50), suggest that fish lymphocytes are able to cross the brain-blood barrier and infiltrate the brain. In fact, though microglial cells are the innate immune cells of the central nervous system, particular populations of T cells, different to the circulating ones, have been identified in mammals (51). In fact, active specific cytotoxic CD8^+^ T/GzmB^+^ cells are detected into the brain in patients under nervous system disorders (52). Whatever the reason, GzmB is produced and released in the seabream brain upon NNV infection, suggesting a greater local CMC activity, which could be a key factor for NNV clearance observed in seabream (28, 53–55). How this occurs and whether this also happens in the sea bass brain merits further investigation.

In conclusion, we have identified complete or partial gene sequences coding for gilthead seabream and European sea bass GzmA, GzmB, and GzmM as evidenced by the conservation of key residues and/or the phylogenetic study. In isolated leucocytes we demonstrated the presence of tryptase, metase, chymase, and very low aspartase activities, which might be related to the human GzmA/K, GzmM, GzmH, and GzmB, respectively. Moreover, during leucocyte innate CMC response *in vitro* against fish target cells, alone or NNV-infected, the granzyme transcription and activity profiles strongly suggest that the CMC is mainly mediated by GzmA/K, showing very low implication of GzmB. During *in vivo* infection with NNV *gzma* gene expression was up-regulated in both fish species suggesting again a major role in the fish CMC activity to clear virus-infected cells. In addition, *gzmb* transcription was up-regulated upon NNV infection only in the sea bass; however, the increase in the presence of GzmB and its distribution in the seabream brain upon NNV infection suggests an important role in the local clearance of the virus, in sharp contrast to the *in vitro* observations. Further studies are needed to establish the mechanisms that orchestrate the presence, production, regulation, secretion, and action mechanisms of the fish granzymes.

## Data Availability Statement

The raw data supporting the conclusions of this manuscript will be made available by the authors, without undue reservation, to any qualified researcher.

## Ethics Statement

Animal handling and sampling was approved by the Bioethical Committees of the IEO and the University of Murcia (Permit Number: A13150104).

## Author Contributions

EC-P, AC, and ME conceived and funded the study and wrote the manuscript. AC performed the *in vitro* CMC assays and granzyme activities. YV performed the *in vivo* study and gene expression. LM and VC performed the gene characterization and *in vitro* gene expression. ML, EC-P, and PR-C carried out the immunohistochemistry.

### Conflict of Interest

The authors declare that the research was conducted in the absence of any commercial or financial relationships that could be construed as a potential conflict of interest.
